# Editorial: Artificial intelligence in multi-omics: advancing tumor metastasis prediction and mechanism analysis

**DOI:** 10.3389/fcell.2026.1924805

**Published:** 2026-07-29

**Authors:** Kun Jiao, Xuexin Li

**Affiliations:** 1 Department of General Surgery, The Fourth Affiliated Hospital, China Medical University, Shenyang, Liaoning, China; 2 Department of Physiology and Pharmacology and Center for Molecular Medicine, Karolinska Institutet and University Hospital, Stockholm, Sweden

**Keywords:** artificial intelligence, multi-omics, precision oncology, single-cell atlases, tumor metastasis

The rapid development of multi-omics technologies has transformed our understanding of tumor metastatic progression (Buissant Des Amorie et al., 2026). Recent advances in cancer biology have shifted the focus from viewing tumors as homogeneous populations toward understanding them as dynamic ecosystems composed of diverse cellular states and molecular interactions. Artificial intelligence (AI) provides powerful computational frameworks for analyzing these complex datasets, enabling more accurate prediction of tumor progression, metastasis and clinical outcomes, aims to highlight cutting-edge methodologies that combine machine learning, deep learning and systems biology with multi-omics datasets (genomics, transcriptomics, proteomics, radiomics, and clinical phenotypes) (Liu et al., 2026). We focus on how AI-driven frameworks can improve metastasis prediction, uncover hidden biological mechanisms and support precision oncology.

Across the six submitted manuscripts, the core goal is to improve the prediction ability of tumor (or blood system malignant tumor) progression, recurrence and survival outcome through machine learning and deep learning methods, and to further explore the key biological factors affecting the progression of the disease Jiao and Li.

Single-cell technologies have fundamentally changed our understanding of tumor heterogeneity by resolving cellular states beyond bulk measurements. Large-scale human cell atlases, including healthy tissue references and disease-associated datasets, provide essential frameworks for comparing normal and malignant cellular programs (Wang et al., 2025). They fundamentally reshape how tumor heterogeneity, metastasis and prognosis are illstrated at both cellular and systems levels (Yu et al., 2025).

The emergence of healthy and tumor tissue single-cell atlases drives this transformation, providing high-resolution views of cell composition, lineage hierarchies and dynamic transcriptional states in the tumor microenvironment, and allowing direct comparisons against reference atlases of healthy tissues (Pan et al., 2023a; 2024; Yang et al., 2024). Besides, the development of tumor subtypes, tumor stem cell-like populations and cross-tumor metastasis are also attributed to single-cell atlas (Chen et al., 2024; Li et al., 2024).

In recent years, spatial transcriptomics has enabled the simultaneous acquisition of gene expression information while preserving tissue spatial architecture, thereby facilitating high-resolution dissection of the tumor microenvironment and progressively emerging as a critical tool for studying tumor metastasis. Researchers are able to systematically reveal the interaction networks between tumor cells and surrounding immune cells, stromal cells and the vascular system, to elucidate the formation process of the pre-metastatic niche, as well as the dynamic remodeling mechanisms of the tumor microenvironment during disease progression (Li H. J. et al., 2025; Qian et al., 2025). For example, cancer-associated fibroblasts (CAFs) actively modulate immunity by remodeling macrophage phenotypes, whereas tumor-associated macrophages (TAMs) contribute to immunosuppression and angiogenesis, thereby promoting metastatic progression (Chen F. et al., 2025; Tang et al., 2026).

Building upon these cellular and spatial maps, multi-scale tumor atlases are emerging as powerful resources for reconstructing metastatic evolution (Terekhanova et al., 2023). These atlases range from human cell reference maps, such as HTCA, to Tumor Ecosystem Atlases and Metastatic Atlases that capture tumor progression across tissues (Pan et al., 2023b; Hong et al., 2026; Cheng et al., 2024). These atlas resources with artificial intelligence enables the extraction of biologically meaningful patterns from high-dimensional data, facilitating the identification of metastatic drivers, reconstruction of evolutionary trajectories and development of prognosis and treatment models for oncology (Cho et al., 2026). These multi-scale maps not only systematically portray the whole process of tumor metastasis, but also provide an important data basis for artificial intelligence-driven biomarker discovery, metastasis mechanism analysis and clinical prognosis prediction. Based on multi-group atlases and advanced machine learning algorithms, researchers are expected to accurately predict the risk of tumor metastasis and further promote the development of precision oncology and individualized treatment (Li et al., 2026).

The rapid and cross-domain expansion of multi-omics technologies has generated unprecedented volumes of biological data, encompassing genomic, transcriptomic, proteomic, metabolomic and spatial information. However, these datasets are characterized by high dimensionality, batch effects and multimodal heterogeneity, posing significant challenges for conventional statistical approaches (Li, 2023; Airoldi et al., 2025). To address these Research Topic, artificial intelligence (AI) has emerged as a powerful analytical paradigm capable of integrating complex biological information and extracting meaningful patterns associated with tumor progression and metastasis (Yates and Van Allen, 2025).

Recent advances in computational frameworks have greatly facilitated the analysis of large-scale single-cell and multi-omics datasets (Yiu et al., 2025). Automated platforms such as Ursa and Cell Analyst streamline key analytical procedures, including data preprocessing, cellular annotation, trajectory inference and functional interpretation, thereby improving the reproducibility and scalability of multi-omics research. These tools have contributed to the construction of comprehensive tumor atlases and have accelerated the identification of metastasis-associated cellular populations and molecular programs (Pan et al., 2023a; 2025).

Beyond dedicated analytical pipelines, the emergence of biological foundation models has further expanded the capabilities of AI in oncology. Models such as scGPT, Geneformer, and BioBERT leverage large-scale biological datasets to learn transferable representations of genes, cells and molecular networks. These approaches enable cross-omics integration, prediction of cellular state transitions and mechanistic interpretation of complex biological processes, providing new opportunities for understanding metastatic evolution (Qiu et al., 2025; Chen Q. et al., 2025).

AI has also become an indispensable tool for predicting tumor metastasis and clinical outcomes. Machine learning algorithms, including Random Forest, XGBoost and Support Vector Machines, have demonstrated strong performance in risk stratification, survival analysis and recurrence prediction (Dai et al., 2025; Xiao et al., 2025; Li X. et al., 2025). More recently, deep learning architectures such as graph neural networks (GNNs), Transformers and multimodal learning frameworks have enabled the simultaneous integration of omics, imaging and clinical data, resulting in more accurate and robust predictive models (Ali et al., 2025; Shen et al., 2025).

Looking forward, the convergence of AI, multi-omics technologies and single-cell atlases is expected to transform precision oncology (Xia et al., 2026). By leveraging comprehensive molecular profiles and predictive modeling, AI can support the identification of metastatic drivers, patient risk stratification, treatment response prediction and personalized therapeutic decision-making. Despite challenges related to data standardization, model interpretability, multicenter validation and clinical implementation, the continued integration of AI and multi-omics is poised to reshape our understanding of tumor metastasis and advance the next-generation of precision cancer medicine (Yiu et al., 2025).

## References

[B1] Airoldi M. Remori V. Fasano M. (2025). Statistical methods for multi-omics analysis in neurodevelopmental disorders: from high dimensionality to mechanistic insight. Biomolecules 15 (10), 1401. 10.3390/biom15101401 41154630 PMC12562612

[B2] Ali M. Richter S. Ertürk A. Fischer D. S. Theis F. J. (2025). Graph neural networks learn emergent tissue properties from spatial molecular profiles. Nat. Commun. 16 (1), 8419. 10.1038/s41467-025-63758-8 40998830 PMC12462520

[B3] Buissant des Amorie J. R. Hageman J. H. Brunner S. R. van der Horst S. E. M. Puschhof M. C. van Hoeck A. (2026). Emergence of oncofetal plasticity is ubiquitous in early colorectal cancers. Nature 654 (8117), 229–239. 10.1038/s41586-026-10344-7 41986711 PMC13233332

[B4] Chen Y. Su Y. Cao X. Siavelis I. Leo I. R. Zeng J. (2024). Molecular profiling defines three subtypes of synovial sarcoma. Adv. Sci. (Weinh). 11 (41), e2404510. 10.1002/advs.202404510 39257029 PMC11892499

[B5] Chen F. Bai G. Liu Q. He G. Ding Z. Liang J. (2025). Integrated multi-omics identifies a CD54+ iCAF-ITGAL+ macrophage niche driving immunosuppression *via* CXCL8-PDL1 axis in cervical cancer. Mol. Cancer 24 (1), 262. 10.1186/s12943-025-02471-y 41121145 PMC12539134

[B6] Chen Q. Hu Y. Peng X. Xie Q. Jin Q. Gilson A. (2025). Benchmarking large language models for biomedical natural language processing applications and recommendations. Nat. Commun. 16 (1), 3280. 10.1038/s41467-025-56989-2 40188094 PMC11972378

[B7] Cheng X. Dai E. Wu J. Flores N. M. Chu Y. Wang R. (2024). Atlas of metastatic gastric cancer links ferroptosis to disease progression and immunotherapy response. Gastroenterology 167 (7), 1345–1357. 10.1053/j.gastro.2024.07.038 39097198

[B8] ChoK. S. LiuY. PeiG. ChenJ. DaiY. LiuY. (2026). Pan-cancer spatial atlas of tertiary lymphoid structures. Science 392 (6801), eadz2742. 10.1126/science.adz2742 42207882

[B9] Dai Z. Shan X. Kang Y. Chen Y. Feng Q. Cai Z. (2025). A multi-omics pipeline integrating machine learning and spatial-cellular analysis identifies SASH1 as a prognostic biomarker and therapeutic target in head and neck squamous cell carcinoma. Int. J. Surg. 111 (12), 9178–9195. 10.1097/JS9.0000000000003647 41099090 PMC12695206

[B10] Hong L. Mei J. Sun X. Wu Y. Dong Z. Jin Y. (2026). Spatial single-cell proteomics landscape decodes the tumor microenvironmental ecosystem of intrahepatic cholangiocarcinoma. Hepatology 83 (1), 57–74. 10.1097/HEP.0000000000001283 39999448

[B11] Li (2023). Harnessing the potential of spatial multiomics: a timely opportunity. Signal Transduct. Target. Ther. 8 (1), 234. 10.1038/s41392-023-01507-3 37302998 PMC10258193

[B12] Li R. Liu X. Huang X. Zhang D. Chen Z. Zhang J. (2024). Single-cell transcriptomic analysis deciphers heterogenous cancer stem-like cells in colorectal cancer and their organ-specific metastasis. Gut 73 (3), 470–484. 10.1136/gutjnl-2023-330243 38050068 PMC10894846

[B13] Li H. J. Qiu Z. B. Wang M. M. Zhang C. Hong H. Z. Fu R. (2025). Radiomics-based support vector machine distinguishes molecular events driving the progression of lung adenocarcinoma. J. Thorac. Oncol. 20 (1), 52–64. 10.1016/j.jtho.2024.09.1431 39306192

[B14] Li X. Pan L. Li W. Liu B. Xiao C. Chew V. (2025). Deciphering immune predictors of immunotherapy response: a multiomics approach at the pan-cancer level. Cell Rep. Med. 6 (4), 101992. 10.1016/j.xcrm.2025.101992 40054456 PMC12047473

[B15] Li L. Zhao W. Zhang Q. Ching W. K. Liu Z. P. (2026). CNet-Cox for interpretable network biomarker discovery and survival risk scoring in precise breast cancer prognosis. NPJ Digit. Med. 10.1038/s41746-026-02756-6 PMC13623954 42185445

[B16] Liu F. Beck S. Yang L. Luo H. Zhang K. (2026). Advancing AI for multi-omics and clinical data integration in basic and translational cancer research. Nat. Rev. Cancer 26 (7), 497–512. 10.1038/s41568-026-00922-2 42014628

[B17] Pan L. Mou T. Huang Y. Hong W. Yu M. Li X. (2023a). Ursa: a comprehensive multiomics toolbox for high-throughput single-cell analysis. Mol. Biol. Evol. 40 (12), msad267. 10.1093/molbev/msad267 38091963 PMC10752348

[B18] Pan L. Shan S. Tremmel R. Li W. Liao Z. Shi H. (2023b). HTCA: a database with an in-depth characterization of the single-cell human transcriptome. Nucleic Acids Res. 51 (D1), D1019–D1028. 10.1093/nar/gkac791 36130266 PMC9825435

[B19] Pan L. Parini P. Tremmel R. Loscalzo J. Lauschke V. M. Maron B. A. (2024). Single cell atlas: a single-cell multi-omics human cell encyclopedia. Genome Biol. 25 (1), 104. 10.1186/s13059-024-03246-2 38641842 PMC11027364

[B20] Pan L. Tang B. Zhang X. Parini P. Tremmel R. Loscalzo J. (2025). Comprehensive analysis of multi-omics single-cell data using the single-cell analyst. iMeta 4 (3), e70038. 10.1002/imt2.70038 40469516 PMC12130572

[B21] Qian J. Shao X. Bao H. Fang Y. Guo W. Li C. (2025). Identification and characterization of cell niches in tissue from spatial omics data at single-cell resolution. Nat. Commun. 16 (1), 1693. 10.1038/s41467-025-57029-9 39956823 PMC11830827

[B22] Qiu P. Chen Q. Qin H. Fang S. Zhang Y. Zhang Y. (2025). BioLLM: a standardized framework for integrating and benchmarking single-cell foundation models. Patterns (N.Y.) 6 (8), 101326. 10.1016/j.patter.2025.101326 40843339 PMC12365531

[B23] Shen P. Yang Z. Sun J. Wang Y. Qiu C. Wang Y. (2025). Explainable multimodal deep learning for predicting thyroid cancer lateral lymph node metastasis using ultrasound imaging. Nat. Commun. 16 (1), 7052. 10.1038/s41467-025-62042-z 40750786 PMC12316972

[B24] Tang J. Chen Z. Zhang D. Jin K. Xu H. Tian C. (2026). ENO2 drives tumor cell-induced M2 macrophage polarization to promote colorectal cancer liver metastasis. Signal Transduct. Target. Ther. 11 (1), 166. 10.1038/s41392-026-02732-2 42082451 PMC13139463

[B25] Terekhanova N. V. Karpova A. Liang W. W. Strzalkowski A. Chen S. Li Y. (2023). Epigenetic regulation during cancer transitions across 11 tumour types. Nature 623 (7986), 432–441. 10.1038/s41586-023-06682-5 37914932 PMC10632147

[B26] Wang Q. Ni Y. Lu S. Zhang B. Ji J. Cai Q. (2025). Multi-dimensional omics integrated machine learning framework identifies macrophage-fibroblast-tumor co-infiltration patterns to predict prognosis in gastric cancer. NPJ Digit. Med. 9 (1), 10. 10.1038/s41746-025-02179-9 41360923 PMC12775079

[B27] Xia P. Shuang S. Fu D. Liu L. Yang D. Guo Y. (2026). Large-scale single-cell analysis and *in silico* perturbation reveal dynamic evolution of HCC: from initiation to therapeutic targeting. NPJ Precis. Oncol. 10 (1), 100. 10.1038/s41698-026-01307-2 41617928 PMC12961043

[B28] Xiao G. Xie R. Gu J. Huang Y. Ding M. Shen D. (2025). Single-cell RNA-Sequencing and spatial transcriptomic analysis reveal a distinct population of APOE-cells yielding pathological lymph node metastasis in papillary thyroid cancer. Clin. Transl. Med. 15 (1), e70172. 10.1002/ctm2.70172 39810624 PMC11733439

[B29] Yang Y. Chen X. Pan J. Ning H. Zhang Y. Bo Y. (2024). Pan-cancer single-cell dissection reveals phenotypically distinct B cell subtypes. Cell 187 (17), 4790–4811.e22. 10.1016/j.cell.2024.06.038 39047727

[B30] Yates J. Van Allen E. M. (2025). New horizons at the interface of artificial intelligence and translational cancer research. Cancer Cell 43 (4), 708–727. 10.1016/j.ccell.2025.03.018 40233719 PMC12007700

[B31] Yiu T. Chen B. Wang H. Feng G. Fu Q. Hu H. (2025). Transformative advances in single-cell omics: a comprehensive review of foundation models, multimodal integration and computational ecosystems. J. Transl. Med. 23 (1), 1176. 10.1186/s12967-025-07091-0 41146276 PMC12560279

[B32] Yu Y. Ouyang W. Huang Y. Huang H. Wang Z. Jia X. (2025). Artificial intelligence-based multi-modal multi-tasks analysis reveals tumor molecular heterogeneity, predicts preoperative lymph node metastasis and prognosis in papillary thyroid carcinoma: a retrospective study. Int. J. Surg. 111 (1), 839–856. 10.1097/JS9.0000000000001875 38990290 PMC11745641

